# Correcting for enzyme immunoassay changes in long term monitoring studies

**DOI:** 10.1016/j.mex.2021.101212

**Published:** 2021-01-06

**Authors:** Abbey E. Wilson, Agnieszka Sergiel, Nuria Selva, Jon E. Swenson, Andreas Zedrosser, Gordon Stenhouse, David M. Janz

**Affiliations:** a Department of Veterinary Biomedical Sciences, University of Saskatchewan, 44 Campus Drive, Saskatoon, SK S7N 5B3, Canada; b Institute of Nature Conservation, Polish Academy of Sciences, Adama Mickiewicza 33, 31120 Krakow, Poland; c Faculty of Environmental Science and Natural Resource Management, Norwegian University of Life Sciences, Høgskoleveien 12, NO-1432, Norway; d Department of Natural Sciences and Environmental Health, University of South-Eastern Norway, Gullbringvegen 36, 3800 Bø, Norway; e Institute for Wildlife Biology and Game Management, University for Natural Resources and Life Sciences, Gregor-Mendel-Straße 33, 1180 Vienna, Austria; f fRI Research, Grizzly Bear Program, 1176 Switzer Drive, Hinton T7V 1V3, AB, Canada

**Keywords:** Hair cortisol, Wildlife, Brown bear, Enzyme immunoassay, Standardization

## Abstract

Enzyme immunoassays (EIAs) are a common tool for measuring steroid hormones in wildlife due to their low cost, commercial availability, and rapid results. Testing technologies improve continuously, sometimes requiring changes in protocols or crucial assay components. Antibody replacement between EIA kits can cause differences in EIA sensitivity, which can hinder monitoring hormone concentration over time. The antibody in a common cortisol EIA kit used for long-term monitoring of stress in wildlife was replaced in 2014, causing differences in cross reactivity and standard curve concentrations. Therefore, the objective of this study was to develop a method to standardize results following changes in EIA sensitivity. We validated this method using cortisol concentrations measured in the hair of brown bears (*Ursus arctos*).

• We used a simple linear regression to model the relationship between cortisol concentrations using kit 1 and kit 2.
• We found a linear relationship between the two kits (R^2^ = 0.85) and used the regression equation (kit2 = (0.98 × kit1) + 1.65) to predict cortisol concentrations in re-measured samples.
• Mean predicted percent error was 16% and 72% of samples had a predicted percent error <20%, suggesting that this method is well-suited for correcting changes in EIA sensitivity.

## Graphical abstract

**Figure:**
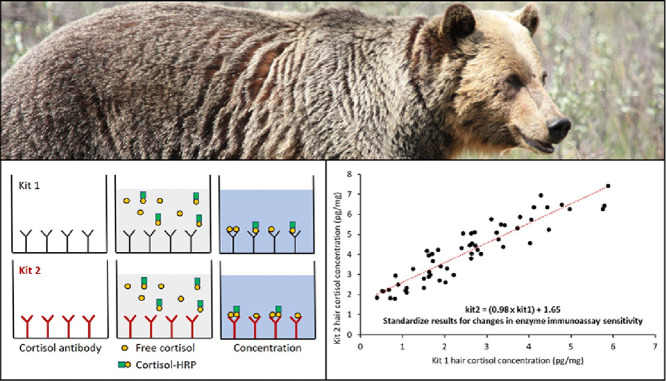

Specifications table

**Table:** 

Subject Area:	Agricultural and Biological Sciences
More specific subject area:	Animal Physiology and Endocrinology
Method name:	Correcting for enzyme immunoassay variation
Name and reference of original method:	N/A
Resource availability:	N/A

## Method details

### Background

Enzyme immunoassays (EIAs) are a common tool for measuring the concentration of steroid hormones in wildlife due to their low cost, commercial availability, and rapid results compared to other approaches [1]. Commercially available EIA kits provide laboratories with easy-to-follow procedures and the majority of materials needed to complete the assay. Oxford Biomedical Research (Oxford, Michigan, USA) manufactures a commonly used EIA kit for the hormone cortisol (EA65 Cortisol EIA kit), which has been used to measure cortisol concentration in the hair of wildlife [2], [3], [4], [5], [6]. The use of this EIA kit has previously been validated in our laboratory for the quantification of cortisol in wildlife hair, and has shown excellent performance characteristics (accuracy and precision) [2,^3^,7]. In competitive EIAs specifically, the analytes within biological samples compete with a known amount of tracer (in this case, cortisol conjugated to horseradish peroxidase (HRP)) for binding to a polyclonal antibody [8]. The HRP activity results in color development, which is read using a spectrophotometer and expressed as optical density. The response is quantified by the amount of cortisol-HRP bound and the amount of unconjugated cortisol in the samples and standards. The concentration of cortisol in samples is then determined by a calibration curve with known concentrations of standards (see supplementary material 1 and 2).

**Fig. 1:**
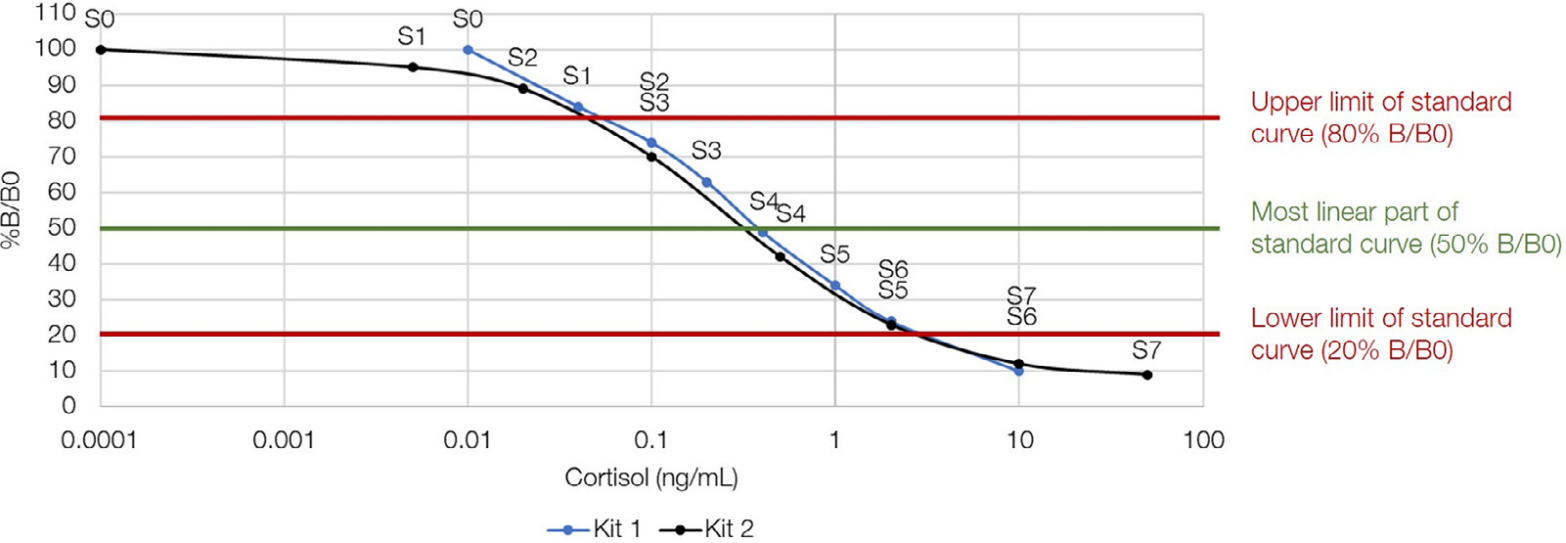
Standard curve cortisol concentrations and percentage bound (%B/B0) for enzyme immunoassay (EIA) kit 1 and kit 2 by Oxford Biomedical Research (Oxford, Michigan, USA). The antibody was replaced in kit 2, resulting in a wider reading frame compared to kit 1. These EIA kits were used to measure cortisol concentration in the hair of brown bears (*Ursus arctos*) collected from free-ranging populations in Sweden and Alberta, Canada from 1996–2013.

As technology continues to develop, laboratories are often faced with changes to protocols, such as antibody replacement or changes in the concentration of antibody, which can lead to differences in EIA sensitivity [9], [10], [11]. These changes become especially important when monitoring hormone concentrations in many individuals or populations over long periods of time (i.e., years to decades), which is often the goal in wildlife studies [6]. The antibody for the cortisol EIA kit by Oxford Biomedical was replaced in 2014, which resulted in differences in cross reactivity and standard curve concentrations compared to the previous antibody (Table 1). A competitive EIA is required to measure cortisol, because it is a small molecule with only one antibody binding site; therefore, the higher the concentration of free cortisol in the sample, the lower the percent bound or absorbance [8]. The sample absorbances can be compared to the B0 well, a blank well that contains no cortisol, in order to obtain the percentage bound (%B/B0). The most accurate concentrations of cortisol in samples are calculated from within the most linear limits of the standard curve. The standard curve is most linear at 50% B/B0, with an upper limit of 80% B/B0 and a lower limit of 20% B/B0 (Fig. 1). The new kit (kit 2) was designed to have a wider reading frame (Table 1), which leads to wide variation in cortisol concentrations when comparing the two kits, as the absorbance results become further away from the 50% B/B0 point on the standard curve (Fig. 1). Therefore, as the results move away from 50% B/B0, small optical density changes return large calculated concentration changes.

**Table 1:** Changes in cross reactivity (A) and standard curve cortisol concentrations (B) between enzyme immunoassay (EIA) kit 1 and kit 2 due to antibody replacement by Oxford Biomedical Research (Oxford, Michigan, USA). These EIA kits were used to measure cortisol concentration in the hair of brown bears (*Ursus arctos*) collected from free-ranging populations in Sweden and Alberta, Canada from 1996–2013.

(A)			(B)		
Cross Reactivity (%)	Kit 1	Kit 2	Standard Curve (Cortisol concentration ng/mL)	Kit 1	Kit 2
Cortisol	100.00	100.00	S0	0.00	0.000
Prednisolone	47.42	66.90	S1	0.04	0.005
Cortisone	15.77	15.90	S2	0.10	0.020
11-deoxycortisol	15.00	58.10	S3	0.20	0.100
prednisone	7.83	13.70	S4	0.40	0.500
corticosterone	4.81	1.40	S5	1.00	2.000
6-b-hydroxycortisol	1.37	3.40	S6	2.00	10.000
17-hydroxyprogesterone	1.36	5.40	S7	10.00	50.000
deoxycorticosterone	0.94	N/A

Given the important role that steroid hormone quantification plays in long-term wildlife monitoring studies, it is crucial to develop a method to account for changes in EIA sensitivity. Therefore, the objective of this study was to present a method to account for changes in EIA sensitivity when measuring hair cortisol concentrations in wildlife with a variety of life history characteristics. Davidian et al. (2015) suggested using pooled control samples to identify differences in EIA accuracy and correcting those changes by fitting a linear regression when measuring fecal glucocorticoid concentrations in free-ranging spotted hyaenas (*Crocuta crocuta*). However, there is often not enough hair to generate a pooled extract to run with each set of samples. Therefore, we modified the existing method and validated it using cortisol concentrations measured in the hair of brown bears (*Ursus arctos*) collected from free-ranging populations monitored by the Scandinavian Brown Bear Research Project (SBBRP) in Sweden and the Foothills Research Institute (fRI Research) in Alberta, Canada. We demonstrate that this method accurately corrects for changes in EIA sensitivity and can be used in long-term monitoring studies.

### Sample collection

A total of 118 hair samples were collected from free-ranging brown bears in Sweden by the SBBRP (*n* = 90 samples from 90 individuals) and in Alberta, Canada (*n* = 28 samples from 28 individuals) from 1996–2013 (Table 2). Hair was either collected by using barbwire snags [6,[12], [13], [14], [15] for population inventory work in Alberta or by plucking with pliers from between the shoulder blades of captured bears in Sweden [16]. Bears were captured by remote drug delivery from a helicopter following procedures as described in Arnemo and Evans (2017) [17]. Following collection, hair samples were placed into envelopes and stored dry at room temperature. These samples were a subset collected for long-term monitoring studies of these populations, which often aim to understand the influence of external factors on concentrations of cortisol in hair at the population level. Therefore, the chosen samples provide an accurate representation of the populations by including different categories of individuals (i.e. sex and age) and common collection methods (i.e. barbed wire snag and plucked over several years). While these attributes may introduce variation within samples, such as unknown sampling location on the body when collected in a barbed wire snag, the chosen samples represent a random assortment of characteristics that are included in our research aims and monitoring programs [18]. All procedures were conducted in accordance with the Alberta Environment and Parks Animal Care Committee, Parks Canada, and the University of Saskatchewan's Committee on Animal Care and Supply for bears sampled in Alberta, Canada, and with the Swedish Environmental Protection Agency, Swedish Board of Agriculture, and Swedish Ethical Committee on Animal Research for bears sampled in Sweden.

**Table 2:** Number of samples for each biological attribute and geographic location. Hair cortisol concentration was measured in brown bears (*Ursus arctos*) using enzyme immunoassay (EIA) kits. A general method was developed to account for changes in EIA sensitivity between kits using these samples.

Geographic location	Year	Sex	Age Class^1^	Collection method
Alberta, Canada	2004: 28	Female: 16 Male: 12	Adult: 11 Subadult: 2 Unknown: 15	Barbed wire snag: 28
Sweden	1996–1999: 26 2000–2005: 53 2006–2013: 11	Female: 45 Male: 45	Adult: 40 Subadult: 50	Plucked: 90

1 Age class was divided into adults (≥5 years old) and sub-adults (<5 years old).

### Cortisol enzyme immunoassay

All cortisol extractions and enzyme immunoassays were completed by the same technician at the University of Saskatchewan. Samples were analyzed using kit 1 in 2011 (May), 2015 (October), and 2016 (February-March) and were re-measured using kit 2 in 2019 (March and August). Cortisol was extracted from brown bear hair as previously described [2,^3^,^5^,19]. We first trimmed all follicles from the hair shaft and used three washes with methanol to remove external contamination of samples (e.g., by blood, feces, soil) [16]. Washed and dried hair was then ground to a fine powder in a mixer mill (Retsch MM400; Retsch GmbH, Germany) and placed on a spinning rotator to extract into methanol for 16–24 h. The samples were then spun in a centrifuge for 15 min (4500 rpm/20 °C) and the supernatant collected and dried under a gentle stream of nitrogen gas for a total of three collections. Concentrated samples were reconstituted with the appropriate volume of buffer solution (10µL/mg). Reconstituted samples were centrifuged to remove any trace hair residue and the supernatant was collected for analysis. We measured the concentration of cortisol (pg/mg) in hair using a validated competitive enzyme-linked immunoassay kit (Oxford EA65 Cortisol EIA kit; Oxford Biomedical Research, Oxford, Michigan, USA), following previously described protocols [2,^3^,^5^,19]. Samples were analyzed undiluted and optical densities were read using a SpectraMAX 190 microplate reader (Molecular Devices, Sunnyvale, California, USA) at 450 nm. A bulk brown bear hair sample was analyzed when possible on a plate, typically once per batch of samples received, as the Oxford Cortisol EIA kit does not supply high and low controls. Intra-assay and inter-assay coefficients of variation were calculated using the bulk extract, and were <15%. Details of additional measures of assay performance (recovery, parallelism, and sensitivity) can be found elsewhere [2,19].

### EIA accuracy

To determine the change in EIA accuracy between kit 1 and kit 2, we first compared the bulk extract (referenced above) run with each sample batch within kit 1 and kit 2. The assay result (ng/mL) of the bulk extract was used to confirm a similar EIA accuracy within each kit as well as a change in EIA accuracy between each kit. By using the bulk extract, we can help eliminate any potential effects of sample characteristics, storage, or extraction on changes in hair cortisol concentration. The bulk extract was initially analyzed using kit 1 in 2016 (February-March), with mean value: $\bar{x}$=0.188±0.023 (SD) ng/mL and range: 0.164 ng/mL-0.215 ng/mL. During the dates of analysis using kit 2 (March and August 2019), the mean value of the bulk extract was $\bar{x}$=0.634±0.020 ng/mL and ranged from 0.619 ng/mL-0.662 ng/mL. Furthermore, the mean B/B0 for the bulk extract analyzed using kit 1 was $\bar{x}$=62.5±2.27%, while the mean B/B0 for the bulk extract analyzed using kit 2 was $\bar{x}$=42.2±0.97%. The inter-assay coefficient of variance (CV) between kit 1 and kit 2 exceeded the commonly accepted limit of 20% (CV=54%), confirming a difference in EIA accuracy between the two kits.

### Data exploration

**Fig. 2:**
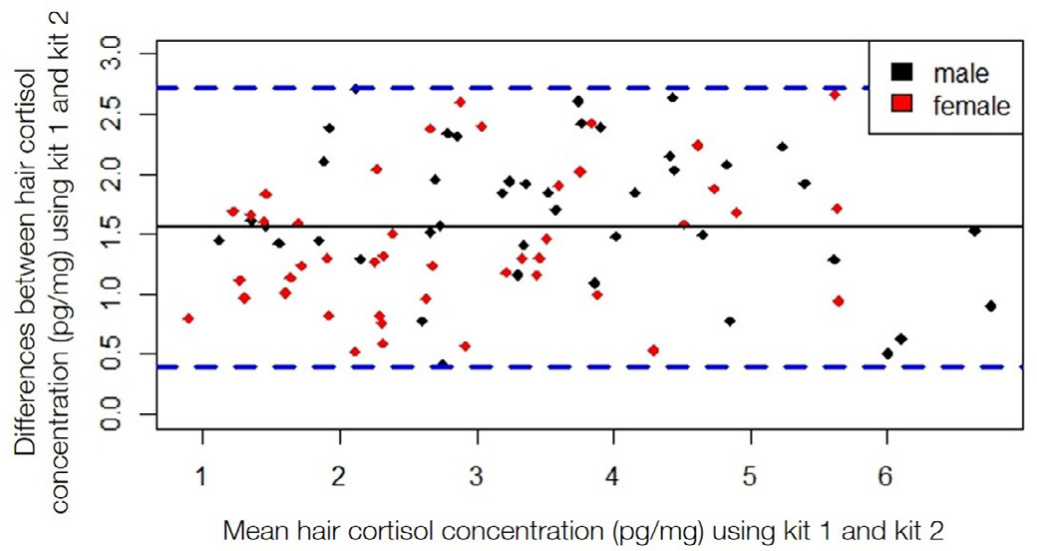
A Bland–Altman plot (difference plot) was used to analyze the agreement between hair cortisol concentrations (HCC) measured by enzyme immunoassay (EIA) kit 1 and kit 2 by Oxford Biomedical Research (Oxford, Michigan, USA). The solid black line is the mean HCC using kit 1 and kit 2. The dashed blue lines represent the 95% limits of agreement for each comparison (mean difference ± 1.96 standard deviation of the difference). Samples that fell outside of the 95% limits of agreement were removed from further analysis (*n* = 25) and the remaining samples (*n* = 86) were used to determine the linear relationship between the two kits. These EIA kits were used to measure cortisol concentration in the hair of brown bears (*Ursus arctos*) collected from free-ranging populations in Sweden and Alberta, Canada from 1996–2013.

For data exploration and normalization, we first removed samples that had a B/B0 <20% and >80% (*n* = 7). Samples that have a B/B0 between 20 and 80% fall within the linear range of the calibration curve and thus provide the most accurate cortisol concentration for calibration purposes between the two kits. While samples were collected by different methods, across years, and in animals with varying life histories, the comparison was between the two kits on the same samples, rather than between samples with different characteristics. Therefore, we used a Bland–Altman plot to analyze the agreement between HCC measured by EIA kit 1 and kit 2 (Fig. 2). Bland-Altman plots are used to compare two methods for clinical assessment by examining the variability of the differences between the two techniques [20,21]. To determine if the two methods agree, samples must fall within the 95% limits of agreement for each comparison (mean difference ± 1.96 standard deviation of the difference) and show no variability of differences between methods and the level of measurement. Samples that fell outside of the 95% limits of agreement were removed, and this process was repeated until all samples fell within these limits (*n* = 86).

To investigate the potential influence of biological attributes (sample year, sex, age class, and collection method) on the difference in HCC measurements, we used a one-way analysis of variance (ANOVA) for each variable on the normally distributed difference values (Shapiro-Wilks test; p = 0.15). Since all samples in Alberta were collected by barbed wire snag and all samples in Sweden were collected by plucking, the effect of geographic area could not be disentangled from the collection method. We calculated the difference in HCC between the two kits by subtracting the measured concentration (pg/mg) of hair cortisol using kit 1 from the measured concentration of hair cortisol using kit 2 for each sample. This allowed us to generate one response variable, with mean $\bar{x}$=1.55±0.59 pg/mg and range: 0.41 pg/mg-2.71 pg/mg, that represented the change between the two kits. The difference in HCC measurements was not significantly (*p* > 0.05) influenced by sample year, age class, or collection method; however, difference values were significantly (*p* = 0.04) influenced by sex (see Supplementary Table 1). Data exploration and normalization methods were completed in Excel software and all statistical analyses were completed in R statistical software (version 3.5.3) [22].

### Method development and validation

**Fig. 3:**
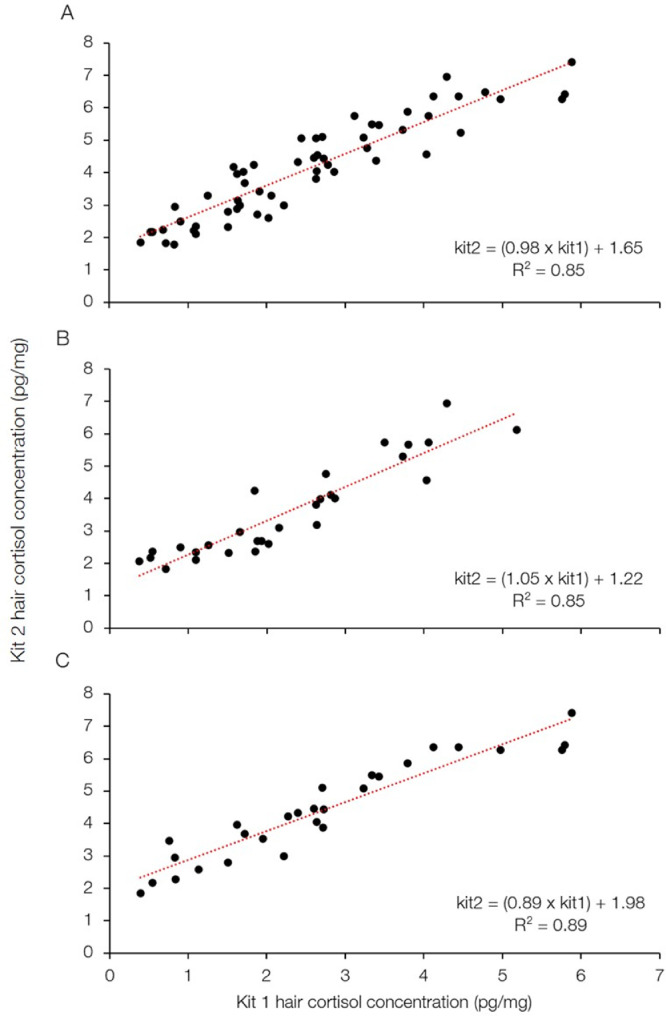
Relationship between hair cortisol concentrations (HCC) measured by enzyme immunoassay (EIA) kit 1 and kit 2 by Oxford Biomedical Research (Oxford, Michigan, USA) in (A) all samples, (B) males only, (C) females only. The antibody was replaced in kit 2, leading to wide variation when comparing HCC using kit 1 vs kit 2. The red dashed line is the simple linear regression fitted to the training samples in order to predict HCC quantified by kit 1. These EIA kits were used to measure cortisol concentration in the hair of brown bears (*Ursus arctos*) collected from free-ranging populations in Sweden and Alberta, Canada from 1996–2013.

To develop a method to standardize the results across the two kits, we used a simple linear regression to model the relationship between the calculated cortisol concentrations obtained using kit 1 versus kit 2. Since there was a significant difference between male and female difference values, the following steps were completed for all data, for males only, and for females only. We first created a mixed population that contained all samples to train and test the predictive model. We randomly assigned 2/3 of the total samples to a train group and 1/3 of the total samples to a test group. We found a linear relationship (kit2 = (0.98 × kit1) + 1.65) with an R^2^ = 0.85 when comparing the calculated cortisol concentrations obtained using kit 1 versus kit 2 from the training group using all data (Fig. 3A) and found similar relationships using males and females only (Fig. 3B-C). These equations were used to predict the hair cortisol concentrations of those samples in the test group for each dataset.

Model performance was assessed by 1) the percent of test samples that were over- or underestimated, 2) the mean (±SD) predicted percent error (((|actual value – predicted value|)/actual value) × 100), 3) the percent of samples with a predicted percent error <20%, and 4) comparison of predicted values and actual matched concentrations using the Wilcoxon's signed-rank test for all data, males only, and females only (Table 3). Based on Davidian et al. (2015), model predictions were considered to be reliable if the difference between predicted values and their matched re-measured values does not exceed 20% and at least 70% of samples have <20% predicted error, as the commonly accepted inter-assay CV is 20%. When considering all samples, the model overestimated the majority of test samples (62% of samples; 18/29 samples) and underestimated 38% of the test samples (11/29 samples). There was a mean predicted percent error (((|actual value – predicted value|)/actual value) × 100) of 16±15% when comparing the predicted concentrations to the actual re-measured concentrations. Furthermore, 72% of test samples (21/29 samples) had a predicted percent error <20%. When data was separated by males and females, the model over- or underestimated roughly half of the test samples. The mean predicted percent error was <20% for both males and females; however, 67% of male samples had a predicted percent error <20%, while 93% of female samples had a predicted percent error <20%. The standardized concentrations predicted using the model were not significantly different from their actual matched concentrations when using all data (predicted median=3.87 pg/mg; actual median=3.52 pg/mg; Wilcoxon's signed-rank test, *V* = 168, *p* = 0.29). Similarly, when using samples collected from males and females separately, there was no significant (*P*>0.05) difference between predicted concentrations and actual matched concentrations (Table 3).

**Table 3:** Relationship between hair cortisol concentrations (HCC) measured by enzyme immunoassay (EIA) kit 1 and kit 2 by Oxford Biomedical Research (Oxford, Michigan, USA) in all samples, males only, females only. Model performance was assessed by over and under estimation of test samples, mean (±SD) predicted percent error (PE; (((|actual value – predicted value|)/actual value) × 100)), percent of samples with a PE<20%, and comparison of predicted values and actual matched concentrations using the Wilcoxon's signed-rank test. These EIA kits were used to measure cortisol concentration in the hair of brown bears (*Ursus arctos*) collected from free-ranging populations in Sweden and Alberta, Canada from 1996–2013.

Data	Equation	R^2^	%over	%under	%PE	%<20%PE	Wilcoxon's signed-rank test (median HCC pg/mg)
All (*n* = 86)	kit2=(0.98 x kit1)+1.65	0.85	62	38	16±15	72	predicted=3.87; actual=3.52; *p* = 0.29
Male (*n* = 44)	kit2=(1.05 x kit1)+1.22	0.85	40	60	14±11	67	predicted=2.93; actual=3.30; *p* = 0.13
Female (*n* = 42)	kit2=(0.89 x kit1)+1.98	0.89	57	43	13±10	93	predicted=4.25; actual=4.59; *p* = 0.95

## Conclusions

We present a method to account for changes in EIA sensitivity when measuring hair cortisol concentrations in wildlife with a variety of life history characteristics. Although other sources of variation, such as sample quality, laboratory conditions, and human error, may play a role in EIA accuracy, this method allows for the long-term monitoring of steroid hormone concentration and subsequent physiological function when changes in EIA kits occur. This method is easy to follow, uses simple statistics, and can be applied to any EIA where sensitivity and/or accuracy has changed between kits, specifically in cases where pooled quality control samples are not feasible. Furthermore, this method saves technician time and laboratory costs by eliminating the need to re-analyze samples. We suggest this method is well-suited for correcting changes in EIA sensitivity, especially for those studies comparing steroid hormone concentration over time.

## Additional information

The quantification of steroid hormones has long been used as a powerful diagnostic and monitoring tool in human medicine [23]. Recently, these techniques have been applied to the non-invasive monitoring of steroid hormones in wildlife for conservation and management purposes [24]. Steroid hormones are typically measured in urine and/or feces, as these samples can be collected non-invasively, and have been quantified in such matrices across a wide array of taxa, including giant anteater (*Myrmecophaga tridactyla*) [25], blue whale (*Balaenoptera musculus*) [26], and tiger (*Panthera tigris*) [27]. The quantification of steroid hormones has become a critical method for monitoring physiological function and responses in wildlife. Monitoring of steroid hormones has been used to determine reproductive state [26], investigate nutritional and health status [28], and measure the stress response to changes in the environment [27].

The measurement of cortisol in hair has been recognized as a reliable biological marker of long-term stress in wildlife [2,^3^,[29], [30], [31]. Nearly all biological functions are influenced by stress and subsequent hypothalamic-pituitary-adrenal (HPA) axis activity, and chronic stress (weeks to months) can lead to suppression of the immune response, poor reproduction, and reduced growth [32]. Cortisol is released following the activation of the HPA axis in response to stress [33]. Free circulating cortisol diffuses into the hair shaft from blood vessels during the active hair growth phase (months to years) [34], making it possible to measure HPA axis activity and cortisol release over time [30]. Furthermore, hair can be collected non-invasively by barbed wire hair snags and/or opportunistically from archives or mortalities, thus eliminating any acute stress due to capture and/or restraint [35]. The measurement of cortisol in hair has become a useful tool to monitor long-term stress in wild animals, especially for those species residing on rapidly changing landscapes [6,14].

The decline in terrestrial vertebrate populations has been, in part, attributed to increased anthropogenic resource use [36]. In particular, brown bears often occupy habitats that are subject to forestry, oil and gas exploration, mining, agriculture, hunting, and other recreational uses [37], [38], [39]. The effects of landscape change on HPA activity are often not included in traditional wildlife monitoring methods; however, anthropogenic disturbance and food availability has been shown to influence long-term stress in brown bears [6]. Stress has also been shown to influence fitness in individual animals [40,41] and thus, the measurement of glucocorticoid concentrations may provide early warning of declining population performance [42,43]. Long-term data sets spanning years to decades are required to measure specific attributes of individual and population performance, such as survival, reproductive output, and abundance. Therefore, consistency in the ability to measure changes in steroid hormone concentration is crucial. Replacement of the antibody or changes in the concentration of the antibody in EIA kits can affect EIA sensitivity, making it difficult to monitor changes in steroid hormone concentration over time. Thus, a method that accounts for changes in EIA kit sensitivity will allow for the long-term monitoring of steroid hormone concentration and subsequent physiological function in wildlife.

## Appendix Supplementary materials

Image, application 1

Image, application 2

Image, application 3
