# Supplementary material for: Correcting for enzyme immunoassay changes in long term monitoring studies
Source: MethodsX. 2021 Jan 6;8:101212. doi: 10.1016/j.mex.2021.101212 (PMC8374155; doi:10.1016/j.mex.2021.101212)
Supplement: Supplementary file 1 [file mmc1.docx]

Supplementary Material

Title: Correcting for enzyme immunoassay changes in long term monitoring studies

Authors: Abbey E. Wilson, Agnieszka Sergiel, Nuria Selva, Jon E Swenson, Andreas Zedrosser, Gordon Stenhouse, David M. Janz

Table 1. A one-way analysis of variance was used to determine the influence of biological attributes (sample year, age class, sex, and collection method) on the difference between hair cortisol concentrations (HCC) measured by enzyme immunoassay (EIA) kit 1 and kit 2 by Oxford Biomedical Research (Oxford, Michigan, USA). These EIA kits were used to measure cortisol concentration in the hair of brown bears (*Ursus arctos*) collected from free-ranging populations in Sweden and Alberta, Canada from 1996-2013.

| **Variable** | **df** | **Sum of Squares** | **Mean of Squares** | **F value** | **Pr(>F)** |
| --- | --- | --- | --- | --- | --- |
| Sample Year (1996-2013) | 12 | 6.04 | 0.50 | 1.54 | 0.13 |
| Residuals | 73 | 23.90 | 0.33 |  |  |
|  |  |  |  |  |  |
| Sex (male and female) | 1 | 1.44 | 1.44 | 4.25 | **0.04** |
| Residuals | 84 | 28.50 | 0.34 |  |  |
|  |  |  |  |  |  |
| Age Class  (adult (≥5 years old) and sub-adult) | 2 | 0.33 | 0.16 | 0.46 | 0.63 |
| Residuals | 83 | 29.62 | 0.36 |  |  |
|  |  |  |  |  |  |
| Collection method  (snagged and plucked) | 1 | 0.40 | 0.40 | 1.15 | 0.29 |
| Residuals | 84 | 29.54 | 0.35 |  |  |
