# Supplementary material for: Correcting for enzyme immunoassay changes in long term monitoring studies
Source: MethodsX. 2021 Jan 6;8:101212. doi: 10.1016/j.mex.2021.101212 (PMC8374155; doi:10.1016/j.mex.2021.101212)
Supplement: Supplementary file 2 [file mmc2.pdf]

Oxford Biomedical Research
SUPERIOR SCIENCE. RELIABLE RESULTS.

Enzyme Immunoassay for
Cortisol
For Research Use Only

INTRODUCTION

Cortisol or hydrocortisone, is the primary corticosteroid secreted by the adrenal cortex. Cortisol is synthesized from cholesterol and may be found in the blood as free cortisol or bound to corticosteroid-binding globulin. The release of cortisol is controlled by ACTH, which is produced in the anterior pituitary. Plasma cortisol levels are highest in the morning and decrease throughout the day. Cortisol concentration in plasma also elevates in response to stress. Cortisol has an anti-inflammatory effect and aids in carbohydrate metabolism, renal function and the promotion of glucogenesis.

Measurement of plasma cortisol level is useful in diagnosing conditions related to functions of the adrenal cortex, including Cushing's syndrome (hypercortisolism), Addison's disease (hypocortisolism) and adrenal tumors. Abnormal cortisol levels may be linked to prostate cancer, depression and schizophrenia.

PRINCIPLES OF PROCEDURE

This is an ELISA (Enzyme-Linked Immunosorbent Assay) for the quantitative analysis of cortisol levels in biological fluid. This test kit operates on the basis of competition between the enzyme conjugate and the cortisol in the sample for a limited number of binding sites on the antibody coated plate.

The sample or standard solution is first added to the micro-plate. Next, the diluted enzyme conjugate is added and the mixture is shaken and incubated at room temperature for one hour. During the incubation, competition for binding sites is taking place. The plate is then washed removing all the unbound material. The bound enzyme conjugate is detected by the addition of substrate that generates an optimal color after 30 minutes. Quantitative test results may be obtained by measuring and comparing the absorbance reading of the wells of the samples against the standards with a microplate reader at 650 nm. The extent of color development is inversely proportional to the amount of cortisol in the sample or standard. For example, the absence of cortisol in the sample will result in a bright blue color, whereas the presence of cortisol will result in decreased or no color development.

MATERIALS PROVIDED

Component	Description	Volume	Storage
**EIA Buffer**	Buffer used to dilute the Conjugate and Cortisol Standards.	30 mL	4°C
**10x Wash Buffer**	Buffer used to wash the plate prior to color development.	20 mL	4°C
**TMB Substrate**	TMB substrate used for color development.	20 mL	4°C
**5x Extraction Buffer**	Buffer used to dilute extracted and non-extracted samples.	30 mL	4°C
**Cortisol-HRP Conjugate**	Cortisol horseradish peroxidase concentrated conjugate.	150 μL	4°C
**Cortisol Standard**	1 μg/mL Cortisol standard solution.	100 μL	4°C
**Coated Plate**	96-well microplate coated with a rabbit anti-cortisol antibody.	1 plate	4°C

MATERIALS NEEDED BUT NOT PROVIDED

1. Microplate reader with a 450 nm or 650 nm filter

2. Adjustable micropipettes (10 – 1000 μL) and tips
3. Deionized water
4. Plate cover or plastic film
5. Test tubes
6. 1 N HCl (optional)

EXTRACTION MATERIALS

1. Ethyl Ether
2. Nitrogen Gas
3. Vortex

STORAGE

1. Store the components of this kit at the temperatures specified on the labels.
2. Unopened reagents are stable until the indicated kit expiration date.
3. Desiccant bag must remain in foil pouch with unused strips. Keep pouch sealed when not in use to maintain a dry environment. Remove excess air before sealing.

WARNINGS AND PRECAUTIONS

1. Use aseptic technique when opening and dispensing reagents.
2. This kit is designed to work properly as provided and instructed. Additions, deletions or substitutions to the procedure or reagents are not recommended, as they may be detrimental to the assay.

PROCEDURAL NOTES

1. The enzyme conjugate is most stable in its concentrated form. Dilute only the volume necessary for the amount of strips currently being used.
2. To minimize errors in absorbance measurements due to handling, wipe the exterior bottom of the microplate wells with a lint-free paper towel prior to inserting into the plate reader.

SAMPLE PREPARATION

1. Urine and tissue culture supernatant can be assayed after diluting them with diluted Extraction Buffer.
2. Plasma and most other mediums will need to be extracted.

EXTRACTION PROTOCOL

1. Pipette 100 μL of plasma into a glass test tube and add 1 mL of Ethyl Ether.
2. Vortex the tube for 30 seconds and allow the phases to separate.
3. Transfer the organic phase into a clean glass test tube and evaporate with a nitrogen stream.
4. Dissolve the residue in 100 μL of diluted Extraction Buffer.
5. Dilute 100 fold by adding 10 μL of the above extract into 990 μL of diluted Extraction Buffer.
6. Vortex and assay 50 μL in duplicates.
7. The values obtained from the assay are multiplied by 100 to give final ng/mL concentrations. If additional dilution is necessary, values must be multiplied by the additional dilution factor in order to calculate final ng/mL concentration.
8. If the concentration is higher than the high range of the standard curve, the samples in #6 need to be further diluted and re-assayed.

REAGENT PREPARATION

1. **5x Extraction Buffer:** Dilute the appropriate amount to 1x with deionized water prior to use.
2. **10x Wash Buffer:** Add 20 mL of 10x Wash Buffer to 180 mL of deionized water prior to use.

3. **Cortisol-HRP Conjugate:** Dilute 110 μL of Conjugate into 5.5 mL total volume of EIA Buffer.

STANDARD CURVE PREPARATION

The Cortisol Standard is provided as a 1 μg/mL stock solution. Use the following tables to dilute a set of standard stock solutions and construct an eight-point standard curve.

Table 1: Standard Stock Preparation

Standard	Cortisol Conc. (ng/mL)	Vol. of EIA Buffer (μL)	Transfer Vol. (μL)	Final Vol. (μL)
A	1000	-	Provided	80
B	20	980	20 μL of A	800
C	2	1800	200 μL of B	1800
D	0.2	1800	200 μL of C	2000

Table 2: Standard Curve Preparation

Standard	Cortisol Conc. (ng/mL)	Vol. of EIA Buffer (μL)	Vol. of Stock B (μL)	Vol. of Stock C (μL)	Vol. of Stock D (μL)
S_0	0	1000	-	-	-
S_1	0.04	800	-	-	200
S_2	0.1	500	-	-	500
S_3	0.2	-	-	-	1000
S_4	0.4	800	-	200	-
S_5	1.0	500	-	500	-
S_6	2.0	-	-	1000	-
S_7	10.0	500	500	-	-

ASSAY PROCEDURE

1. Add 50 μL of Standards or Samples (may require diluting) to the corresponding wells on the microplate in duplicate. See **Scheme I** for a sample plate layout.
2. Add 50 μL of diluted Cortisol-HRP Conjugate to each well. Incubate at room temperature for one hour.
3. Wash the plate three times with 300 μL of diluted Wash Buffer per well. Wash 5 times if using an automated plate washer.
4. Add 150 μL of TMB Substrate to each well. Incubate at room temperature for 30 minutes.
5. Read the plate at 650 nm.
   Alternately, the color reaction can be stopped after 10-15 minutes by adding 50 μL of 1 N HCl and read at 450 nm.
   NOTE: If accounting for substrate background, use 2 wells as blanks (BLK) with only 150 μL TMB Substrate in the wells. Subtract the average of these absorbance values from the absorbance values of the wells being assayed.

Scheme I: Sample Plate Layout

	**1**	**2**	**3**	**4**	**5**	**6**	**7**	**8**	**9**	**10**	**11**	**12**
**A**	S_0	S_0	U_1	U_1	U_9	U_9	U_{17}	U_{17}	U_{25}	U_{25}	U_{33}	U_{33}
**B**	S_1	S_1	U_2	U_2	U_{10}	U_{10}	U_{18}	U_{18}	U_{26}	U_{26}	U_{34}	U_{34}
**C**	S_2	S_2	U_3	U_3	U_{11}	U_{11}	U_{19}	U_{19}	U_{27}	U_{27}	U_{35}	U_{35}
**D**	S_3	S_3	U_4	U_4	U_{12}	U_{12}	U_{20}	U_{20}	U_{28}	U_{28}	U_{36}	U_{36}
**E**	S_4	S_4	U_5	U_5	U_{13}	U_{13}	U_{21}	U_{21}	U_{29}	U_{29}	U_{37}	U_{37}
**F**	S_5	S_5	U_6	U_6	U_{14}	U_{14}	U_{22}	U_{22}	U_{30}	U_{30}	U_{38}	U_{38}

**G**	S_6	S_6	U_7	U_7	U_{15}	U_{15}	U_{23}	U_{23}	U_{31}	U_{31}	U_{39}	U_{39}
**H**	S_7	S_7	U_8	U_8	U_{16}	U_{16}	U_{24}	U_{24}	U_{32}	U_{32}	BLK	BLK

CALCULATIONS

1. After the substrate background has been subtracted from all absorbance values, average all of your duplicate well absorbance values.
2. The average of your two S_0 values is now your B_0 value. (S_1 now becomes B_1, etc.)
3. Next, find the percent of maximal binding (%B/B_0 value). To do this, divide the averages of each standard absorbance value (now known as B_1 through B_7) by the B_0 absorbance value and multiply by 100 to achieve percentages.
4. Graph your standard curve by plotting the %B/B_0 for each standard concentration on the y-axis against concentration on the x-axis. Draw a curve by using a curve-fitting routine (i.e. 4-parameter or linear regression).
5. Divide the averages of each sample absorbance value by the B_0 value and multiply by 100 to achieve percentages.
6. Using the standard curve, the concentration of each sample can be determined by comparing the %B/B_0 of each sample to the corresponding concentration of standard.
7. If the samples were diluted, the concentration determined from the standard curve must be multiplied by the dilution factor.

Figure 1: Typical Standard Curve

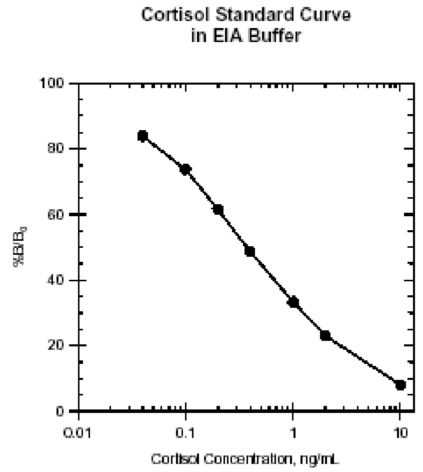

CROSS REACTIVITY

Cortisol	100.00%	Dexamethazone	0.03%
Prednisolone	47.42%	Beclomethazone	0.01%
Cortisone	15.77%	d-Aldosterone	0.01%
11-Deoxycortisol	15.00%	Testosterone	0.01%
Prednisone	7.83%	17α-Hydroxypregnenolone	<0.01%
Corticosterone	4.81%	Androstendione	<0.01%
6-β-Hydroxycortisol	1.37%	Cholesterol	<0.01%
17-Hydroxyprogesterone	1.36%	Estradiol	<0.01%
Deoxycorticosterone	0.94%	Estriol	
Progesterone	0.06%	Estrone	
Betamethasone	0.05%	Pregnenolone	
Dehydroepiandrosterone	0.03%		

DISCLAIMER

This information is believed to be correct but does not purport to be all-inclusive and shall be used only as a guide. Oxford Biomedical Research, Inc. shall not be held liable for any damage resulting from handling or from contact with the above product. See catalog for additional terms and conditions of sale.

ORDERING INFORMATION

For additional kits or a complete catalog please call 800-692-4633.

TECHNICAL SUPPORT

If you need technical information or assistance with assay procedures, call our Technical Support Department at 800-692-4633 or 248-852-8815. Our staff will be happy to answer your questions about this or any other product in the Oxford Biomedical line.

GUARANTEE AND LIMITATION OF REMEDY

Oxford Biomedical Research, Inc. makes no guarantee of any kind, expressed or implied, which extends beyond the description of the materials in this kit, except that these materials and this kit will meet our specifications at the time of delivery. Buyer's remedy and Oxford Biomedical Research, Inc.'s sole liability hereunder is limited to, at Oxford Biomedical Research, Inc.'s option, refund of the purchase price of, or the replacement of, material that does not meet our specification. By acceptance of our products, Buyer indemnifies and holds Oxford Biomedical Research, Inc. harmless against, assumes all liability for the consequence of its use or misuse by the Buyer, its employees, or others. Said refund or replacement is conditioned on Buyer notifying Oxford Biomedical Research, Inc. within thirty (30) days of the receipt of product. Failure of Buyer to give said notice within thirty (30) days of receipt of product shall constitute a waiver by the Buyer of all claims hereunder with respect to said material(s).

Oxford Biomedical Research, Inc.
P.O. Box 522
Oxford, MI 48371 U.S.A.

Orders: 800-692-4633
Technical Service: 248-852-8815
Fax: 248-852-4466
E-mail: info@oxfordbiomed.com

Made in the U.S.A.
