# Supplementary material for: Correcting for enzyme immunoassay changes in long term monitoring studies
Source: MethodsX. 2021 Jan 6;8:101212. doi: 10.1016/j.mex.2021.101212 (PMC8374155; doi:10.1016/j.mex.2021.101212)
Supplement: Supplementary file 3 [file mmc3.pdf]

Enzyme Immunoassay for
Cortisol

For Research Use Only

INTRODUCTION

Cortisol is a glucocorticoid steroid hormone produced in the adrenal cortex of the adrenal gland. Cortisol is produced throughout the day; with very high levels in the early morning it quickly decreases to a low level in the early afternoon. After that, it continues to slowly decrease into the night quickly spiking before morning. Cortisol levels may also spike immediately after waking up, during periods of low blood glucose levels, and in response to stress.

Cortisol is also known as Hydrocortisone when it is used medicinally, usually as a supplement or for its anti-inflammatory properties. Knowing plasma Cortisol levels is useful in identifying a serious lack of cortisol usually found in Addison's disease, or a serious overproduction of cortisol as in Cushing's syndrome. Cortisol is also frequently used to evaluate the stress levels of a subject.

PRINCIPLES OF PROCEDURE

This kit is a competitive enzyme-linked immunoassay (ELISA) for determining levels of Cortisol in biological samples such as urine, saliva, and plasma. Briefly, cortisol in the samples or standards competes with cortisol conjugated to horseradish peroxidase (HRP) for binding to a polyclonal antibody specific for cortisol coated on the microplate. The HRP activity results in brilliant blue color development when the substrate is added, with the intensity of the color proportional to the amount of cortisol-HRP bound and inversely proportional to the amount of unconjugated cortisol in the samples or standards.

MATERIALS PROVIDED

Component	Description	Volume	Storage	Cat. No.
**Coated Plate**	96-well microplate coated with a rabbit anti-cortisol antibody.	1 plate	4°C	EA65a
**Cortisol Standard**	0.5 µg/mL Cortisol standard solution.	100 µL	4°C	EA65b
**EIA Buffer**	Buffer used to dilute the Conjugate and Cortisol Standards.	30 mL	4°C	EA65c
**5x Extraction Buffer**	Buffer used to dilute extracted and non-extracted samples.	30 mL	4°C	EA65d
**10x Wash Buffer**	Buffer used to wash the plate prior to color development.	20 mL	4°C	EA65e
**Cortisol-HRP Conjugate**	Cortisol horseradish peroxidase concentrated conjugate.	150 µL	4°C	EA65f
**TMB Substrate**	TMB substrate used for color development.	20 mL	4°C	EA65g

MATERIALS NEEDED BUT NOT PROVIDED

1. Microplate reader with a 450 nm or 650 nm filter
2. Adjustable micropipettes (10 – 1000 µL) and tips
3. Deionized water
4. Plate cover or plastic film
5. Test tubes
6. 1 N HCl (optional)

 © Oxford Biomedical Research Inc., 2015

EXTRACTION MATERIALS

1. Ethyl Ether
2. Nitrogen Gas
3. Vortex

STORAGE

1. Store the components of this kit at the temperatures specified on the labels.
2. Unopened reagents are stable until the indicated kit expiration date.
3. Desiccant bag must remain in foil pouch with unused strips. Keep pouch sealed when not in use to maintain a dry environment. Remove excess air before sealing.

WARNINGS AND PRECAUTIONS

1. Use aseptic technique when opening and dispensing reagents.
2. This kit is designed to work properly as provided and instructed. Additions, deletions or substitutions to the procedure or reagents are not recommended, as they may be detrimental to the assay.

PROCEDURAL NOTES

1. The enzyme conjugate is most stable in its concentrated form. Dilute only the volume necessary for the amount of strips currently being used.
2. To minimize errors in absorbance measurements due to handling, wipe the exterior bottom of the microplate wells with a lint-free paper towel prior to inserting into the plate reader.

SAMPLE PREPARATION

1. Saliva, urine, and tissue culture supernatant can be assayed after diluting them with diluted Extraction Buffer.
2. Plasma and most other mediums will need to be extracted using the extraction protocol below.

EXTRACTION PROTOCOL

1. Pipette 100 μL of plasma into a glass test tube and add 1 mL of Ethyl Ether.
2. Vortex the tube for 30 seconds and allows the phases to separate.
3. Transfer the upper organic phase into a clean glass test tube and evaporate the solvent using a stream of nitrogen gas. Discard the aqueous phase.
4. Dissolve the residue in 100 μL of diluted Extraction Buffer.
5. Dilute 100 fold by adding 10 μL of the above extract into 990 μL of diluted Extraction Buffer. Store unused sample at -20°C.
6. Vortex the sample and proceed to the assay procedure.
7. The values obtained from the assay are multiplied by 100 to give final ng/mL concentrations. If additional dilution is necessary, values must be multiplied by the additional dilution factor in order to calculate final ng/mL concentration.
8. If the concentration is higher than the high range of the standard curve, the sample should be further diluted. If the concentration is undetectable, reduce the dilution in step 5 above.

REAGENT PREPARATION

1. **5x Extraction Buffer:** Dilute the appropriate amount to 1x with deionized water prior to use.
2. **10x Wash Buffer:** Add 20 mL of 10x Wash Buffer to 180 mL of deionized water prior to use.
3. **Cortisol-HRP Conjugate:** Dilute 110 μL of Conjugate into 5.5 mL total volume of EIA Buffer.

STANDARD CURVE PREPARATION

The Cortisol Standard is provided as a 500 ng/mL stock solution. Use the following table to dilute a set of standard stock solutions and construct an eight-point standard curve.

Table 1: Standard Curve Preparation

Standard	Cortisol Conc. (ng/mL)	Vol. of EIA Buffer (μL)	Transfer Volume (μL)	Transfer Source	Final Volume (μL)
S_7	50	450	50	Cortisol Standard	400
S_6	10	400	100	S_7	400
S_5	2	400	100	S_6	375
S_4	0.5	375	125	S_5	400
S_3	0.1	400	100	S_4	400
S_2	0.02	400	100	S_3	375
S_1	0.005	375	125	S_2	500
S_0	0.000	450	-	-	450

ASSAY PROCEDURE

1. Add 50 μL of Standards or Samples (may require diluting) to the corresponding wells on the microplate in duplicate. See **Scheme I** for a sample plate layout.
2. Add 50 μL of diluted Cortisol-HRP Conjugate to each well. Incubate at room temperature for one hour.
3. Wash the plate three times with 300 μL of diluted Wash Buffer per well. Wash 5 times if using an automated plate washer.
4. Add 150 μL of TMB Substrate to each well. Incubate at room temperature for 30 minutes.
5. Read the plate at 650 nm.
   Alternately, the color reaction can be stopped after 30 minutes by adding 50 μL of 1 N HCl and read at 450 nm.
   NOTE: If accounting for substrate background, use 2 wells as blanks (BLK) with only 150 μL TMB Substrate in the wells. Subtract the average of these absorbance values from the absorbance values of the wells being assayed.

Scheme I: Sample Plate Layout

	1	2	3	4	5	6	7	8	9	10	11	12
**A**	S_0	S_0	U_1	U_1	U_9	U_9	U_{17}	U_{17}	U_{25}	U_{25}	U_{33}	U_{33}
**B**	S_1	S_1	U_2	U_2	U_{10}	U_{10}	U_{18}	U_{18}	U_{26}	U_{26}	U_{34}	U_{34}
**C**	S_2	S_2	U_3	U_3	U_{11}	U_{11}	U_{19}	U_{19}	U_{27}	U_{27}	U_{35}	U_{35}
**D**	S_3	S_3	U_4	U_4	U_{12}	U_{12}	U_{20}	U_{20}	U_{28}	U_{28}	U_{36}	U_{36}
**E**	S_4	S_4	U_5	U_5	U_{13}	U_{13}	U_{21}	U_{21}	U_{29}	U_{29}	U_{37}	U_{37}
**F**	S_5	S_5	U_6	U_6	U_{14}	U_{14}	U_{22}	U_{22}	U_{30}	U_{30}	U_{38}	U_{38}
**G**	S_6	S_6	U_7	U_7	U_{15}	U_{15}	U_{23}	U_{23}	U_{31}	U_{31}	U_{39}	U_{39}
**H**	S_7	S_7	U_8	U_8	U_{16}	U_{16}	U_{24}	U_{24}	U_{32}	U_{32}	BLK	BLK

CALCULATIONS

1. Subtract the substrate blank from all absorbance values and then average all duplicate wells for standards and unknown samples.
2. The average of your two S_0 values is now your B_0 value (S_1 now becomes B_1, etc.). B_0 will be your highest OD and represents the standard without added cortisol.

3. Divide the averaged OD value of each standard (B_1 through B_7) by the B_0 to express it as a percent of maximum binding (%B/B_0).
4. Graph your standard curve by plotting the %B/B_0 for each standard concentration on the y-axis against concentration on the x-axis. Draw a curve by using a curve-fitting routine (i.e. 4-parameter or linear regression).
5. Divide the averages of each sample absorbance value by the B_0 value and multiply by 100 to achieve percentages (%B/B_0).
6. Determine the concentration of each sample by comparing to the standard curve. For best results, sample %B/B_0 should be between 20-80%. Samples falling outside 20-80% B/B_0 should be rediluted and rerun.
7. If the samples were diluted, the concentration determined from the standard curve must be multiplied by the dilution factor. If the samples were extracted, remember to multiply by that dilution value as well.

Figure 1: Typical Standard Curve

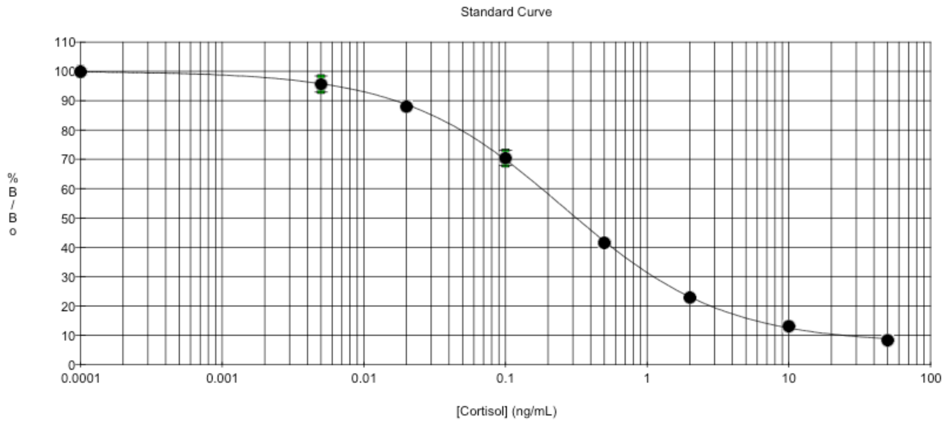

CROSS REACTIVITY

Cortisol	100.00%	Estrone	4.1%
Prednisolone	66.9%	d-Aldosterone	3.6%
11-Deoxycortisol	58.1%	Progesterone	3.5%
Cortisone	15.9%	6-β-Hydroxycortisol	3.4%
Prednisone	13.7%	trans Dehydroandrosterone	1.9%
17-Hydroxyprogesterone	5.4%	Testosterone	1.7%
Dexamethasone	4.6%	Corticosterone	1.4%
Estriol	4.5%	Pregnenolone	1.3%

DISCLAIMER

This information is believed to be correct but does not purport to be all-inclusive and shall be used only as a guide. Oxford Biomedical Research, Inc. shall not be held liable for any damage resulting from handling or from contact with the above product. See catalog for additional terms and conditions of sale.

ORDERING INFORMATION

For additional kits or a complete catalog please call 800-692-4633.

TECHNICAL SUPPORT

If you need technical information or assistance with assay procedures, call our Technical Support Department at 800-692-4633 or 248-852-8815. Our staff will be happy to answer your questions about this or any other product in the Oxford Biomedical line.

GUARANTEE AND LIMITATION OF REMEDY

Oxford Biomedical Research, Inc. makes no guarantee of any kind, expressed or implied, which extends beyond the description of the materials in this kit, except that these materials and this kit will meet our specifications at the time of delivery. Buyer's remedy and Oxford Biomedical Research, Inc.'s sole liability hereunder is limited to, at Oxford Biomedical Research, Inc.'s option, refund of the purchase price of, or the replacement of, material that does not meet our specification. By acceptance of our products, Buyer indemnifies and holds Oxford Biomedical Research, Inc. harmless against, assumes all liability for the consequence of its use or misuse by the Buyer, its employees, or others. Said refund or replacement is conditioned on Buyer notifying Oxford Biomedical Research, Inc. within thirty (30) days of the receipt of product. Failure of Buyer to give said notice within thirty (30) days of receipt of product shall constitute a waiver by the Buyer of all claims hereunder with respect to said material(s).

Oxford Biomedical Research, Inc.
P.O. Box 522
Oxford, MI 48371 U.S.A.

Orders: 800-692-4633
Technical Service: 248-852-8815
Fax: 248-852-4466
E-mail: info@oxfordbiomed.com

Made in the U.S.A.
